# Secretory locations of SIPC in *Amphibalanus amphitrite* cyprids and a novel function of SIPC in biomineralization

**DOI:** 10.1038/srep29376

**Published:** 2016-07-20

**Authors:** Gen Zhang, Xiao-Xue Yang, Pok Man Leung, Li-Sheng He, Tat Yin Chan, Guo-Yong Yan, Yu Zhang, Jin Sun, Ying Xu, Pei-Yuan Qian

**Affiliations:** 1 Environmental Science Programs and Division of Life Science, School of Science, the Hong Kong University of Science and Technology, Clearwater Bay, Kowloon, Hong Kong S.A.R., P. R. China; 2 Sanya Institute of Deep-sea Science and Engineering, Chinese Academy of Science, No. 62, Fenghuang Road, Sanya, Hainan, 572000, P. R. China; 3 Shenzhen Key Laboratory of Marine Bioresource and Eco-environmental Science, College of Life Science, Shenzhen University, Shenzhen, 518060, P. R. China

## Abstract

Settlement-inducing protein complex (SIPC) is a pheromone that triggers conspecific larval settlement in the barnacle *Amphibalanus amphitrite*. In the present study, immunostaining and scanning electron microscopy of SIPC revealed signals in the frontal horn pores and the secretions from carapace pores, suggesting that SIPC might be directly secreted from these organs in *A. amphitrite* cyprids. Further observations showed that the frontal horn pores could contact surfaces while cyprids were “walking”. Immunostaining for SIPC on the contacted surfaces displayed SIPC signals. These signals were similar to the frontal horn pores in size and morphology, suggesting that frontal horn pores might deposit SIPC. Besides, full-length SIPC was expressed and subsequent assays indicated that recombinant SIPC was able to bind to chitins and induce the precipitation of CaCO_3_. Furthermore, recombinant SIPC inhibited the formation of vaterites and regulated the morphology of calcite crystals. The crystals that formed with recombinant SIPC were more stable against water erosion. Overall, these results reported a novel function of recombinant SIPC that regulates crystal formation in barnacle shells.

The barnacle *Amphibalanus amphitrite* is a typical biofouling organism. The life history of *A. amphitrite* consists of four stages, including nauplius, cyprid, juvenile and adult stages^1^.

In the 1990s, a glycoprotein was discovered that induced conspecific larval settlement in the barnacle *A. amphitrite* and was named settlement-inducing protein complex (SIPC)^2^,^3^,^4^. SIPC is most similar to α2-macroglobulin (A2M), demonstrating a sequence similarity of up to 31%^4^. Three subunits of SIPC, corresponding to 98, 88 and 76 kDa, were identified *in vivo*^5^. Among them, the 98 and 88-kDa subunits originated from the N-terminus of SIPC, while the 76-kDa subunit was from the C-terminus^5^.

SIPC is expressed in the cuticles of both barnacle larvae and adults^6^. In nauplius II larvae, SIPC is expressed most strongly in the mouthparts and the hindgut^6^. In cyprids, SIPC is detected in the thoracopods, antennules and carapaces^6^, and it is also found in “footprints” deposited by cyprids on explored surfaces^7^. SIPC is not only present in all of the adult organs that are lined by cuticular tissues, but it is also present in the shell^6^. To date, the known functions of SIPC include the induction of larval settlement as a pheromone^6^,^7^ and the temporary attachment of cyprids as a “sticky” adhesive^8^.

The locations of SIPC secretion during the cyprid stage remain unclear. One hypothesis is that bacteria degrade barnacle cuticles and then release SIPC into the seawater during molting or cuticle regeneration^6^. However, this hypothesis is not completely supported by the available evidence. If a high density of cyprids is transferred to a new tank, the settlement rate increases^9^,^10^,^11^. However, no cuticle molting occurred prior to accomplishing the settlement process in these experiments. Thus, SIPC might be released by cyprids before molting. Another hypothesis is that cyprids secrete SIPC into “footprints” through antennular disks during environmental exploration. This hypothesis is based on the results of SIPC immunostaining, which revealed positive signals on nitrocellulose membranes on which the cyprids had “walked”. These signals were claimed to be cyprid “footprints”^5^,^7^ and were assumed to be secreted by the antennular disks^12^ that have lots of sensory seta^13^,^14^,^15^. However, the secretion of SIPC through antennular disks cannot explain the expression of SIPC in cyprid carapaces. Whether cyprids release SIPC through carapaces remains unknown. High-resolution immunohistochemical studies of SIPC on the body surface of cyprids would aid in resolving this question.

SIPC is highly expressed in barnacle shells^5^. It has been hypothesized that SIPC binds tightly to shell components (such as chitin or minerals), because a strong buffer (containing SDS and DTT) is required to extract SIPC from barnacle shells^16^. Moreover, inhibition of p38 MAPK pathway^17^ and elevation of endogenous nitric oxide concentration^18^ both decrease the expression level of SIPC in cyprids. It is difficult to attribute these results to the two known functions of SIPC (pheromone and adhesive). Polysaccharide chains have been reported to participate in the biomineralization process as nucleators in both vertebrates and invertebrates^19^. Similarly, polysaccharide chains from proteoglycans might participate in shell formation in the barnacle shell by modifying the morphology of calcite crystals^19^,^20^,^21^,^22^. Saccharides derived from glycoproteins and proteoglycans possess many of these same characteristics^23^. As a highly glycosylated protein^4^, SIPC might also function in the biomineralization process in barnacle shells. Moreover, mannose and glucose, which are also the main components of SIPC sugar chains^24^, regulate the morphology of calcite crystals^25^, further supporting the proposed function of SIPC in biomineralization.

In the present study, full-length SIPC was cloned from *A. amphitrite* and polyclonal antibodies against SIPC were generated. Then, we used both fluorescent immunostaining (FI) and immuno-scanning electron microscopy (I-SEM) to identify the localization of SIPC in barnacle shells, cyprid-interacted surfaces and cyprid carapaces. To further investigate the possible function of SIPC in biomineralization, full-length SIPC was expressed using the baculovirus-insect cell expression system. The binding affinity between recombinant SIPC and chitin, as well as the effects of SIPC on CaCO_3_ precipitation and crystallization, were determined. The results of this study extend our understanding in the locations of SIPC secretion in cyprids and the biological functions of SIPC in adult barnacles.

## Results

### SIPC immunostaining in cyprids

**Figure 1:**
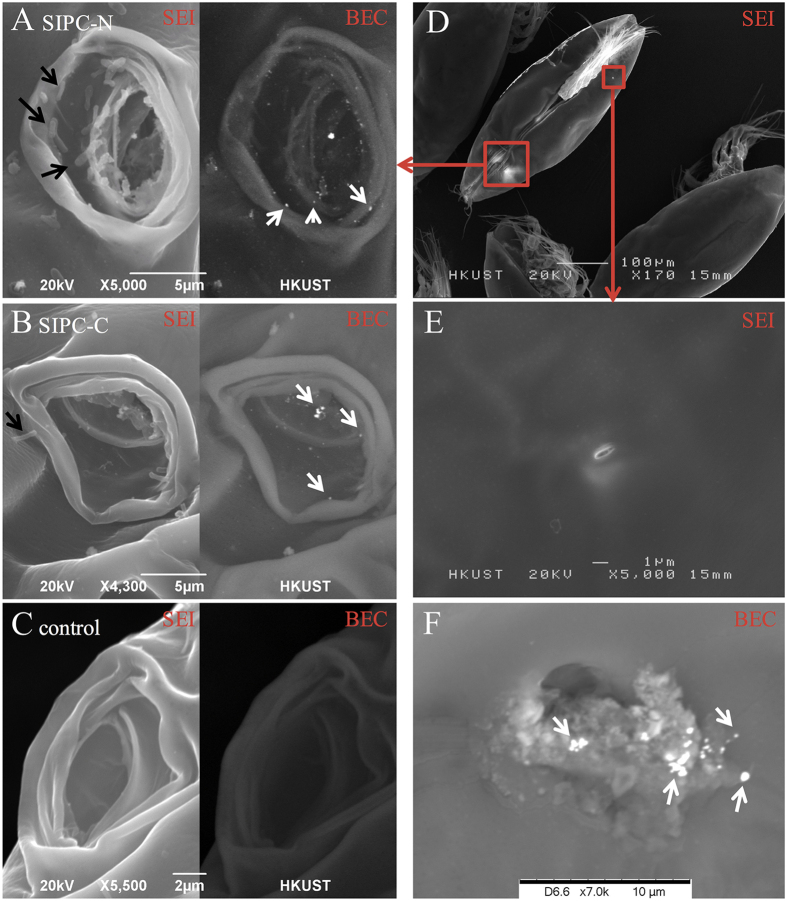
I-SEM studies of SIPC on the body surfaces of cyprids. (**A–C**) show the I-SEM results for the frontal horn pore area using antibodies against SIPC-N terminus, SIPC-C terminus and the control (secondary antibody only), respectively. The left and right portions of the images were obtained using the SEI and BEC models, respectively. In the BEC model, the white dots indicate silver particles, which were observed in the frontal horn pores. The black arrows indicate bacteria-like structure. (**D**) A ventral view of a cyprid showing the position of the paired frontal horn pores and one carapace pore. (**E**) A carapace pore at high magnification. (**F**) A carapace pore releasing secretions. SIPC-immunoreactive signals (white dots) were observed in the secretions. The white arrows indicate silver particles. The I-SEM analysis was repeated for three batches of cyprids with 100 individuals for each. One group of images were presented.

I-SEM for SIPC was performed on cyprids. Under the backscattered electron composition image (BEC) model, silver particles (appearing in white and representing SIPC signals) were localized to the frontal horn pores (Fig. 1A,B) and the secretions from the carapace pores (Fig. 1F). The length of the frontal horn pore and antennular disk was estimated, ranging from 8.8–16.7 μm (13.5 ± 2.5 μm, mean ± SD, n = 8) and 15.3–22.2 μm (18.2 ± 2.4 μm, n = 8), respectively.

FI for SIPC was performed using cyprid sections (Supplementary Fig. S1). Consistent with previous findings, strong signals were observed in the thoraco-abdomen^7^. Relatively weaker SIPC signals were observed at the surface of the cement glands. However, no signals of SIPC were observed in the secretion cells inside the cement glands (Supplementary Fig. S1).

### Fluorescent immunostaining for SIPC on the cyprid-contacted surface

**Figure 2:**
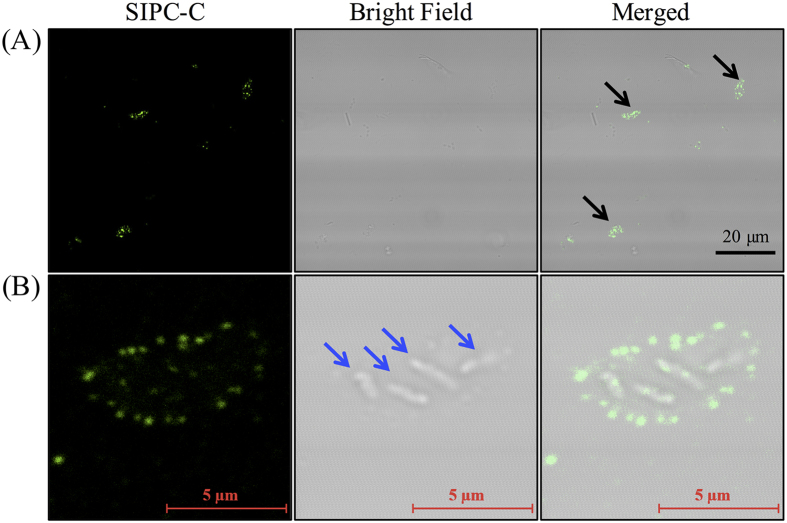
Fluorescent immunostaining for SIPC on cyprid-contacted surfaces. (**A**) The black arrows indicate SIPC-immunoreactive signals in cyprid deposits. (**B**) A view of one cyprid deposit at high magnification. The blue arrows point to the rod-like structures that were considered to be bacteria. The immunostaining was repeated three times and one group of images were presented. The 3D view of a cyprid deposit was constructed using Z-stack analysis and processed in IMARIS (Supplementary Movie S1).

The FI studies revealed that the SIPC-immunoreactive deposits on the glass coverslips were shaped like crescents or triangles (Fig. 2). The length of the deposits ranged from 5.0 to 10.0 μm (6.9 ± 1.8 μm, mean ± SD, n = 6) (Fig. 2). Bright field microscopy observations revealed several rod-like structures in the center of the deposits that could be bacteria. The Z-stack images of the SIPC-immunoreactive deposits were observed using the confocal microscope. Next, the 3D structure of the deposits were constructed from these Z-stack images. Based on the 3D observation, SIPC-immunoreactive deposits were hill-like in shape, with the central region thicker than the edges (Supplementary Movie S1). SIPC signals was uniformly and widely distributed on the surface of the deposits (Supplementary Movie S1).

### Immunostaining for SIPC in barnacle shells

**Figure 3:**
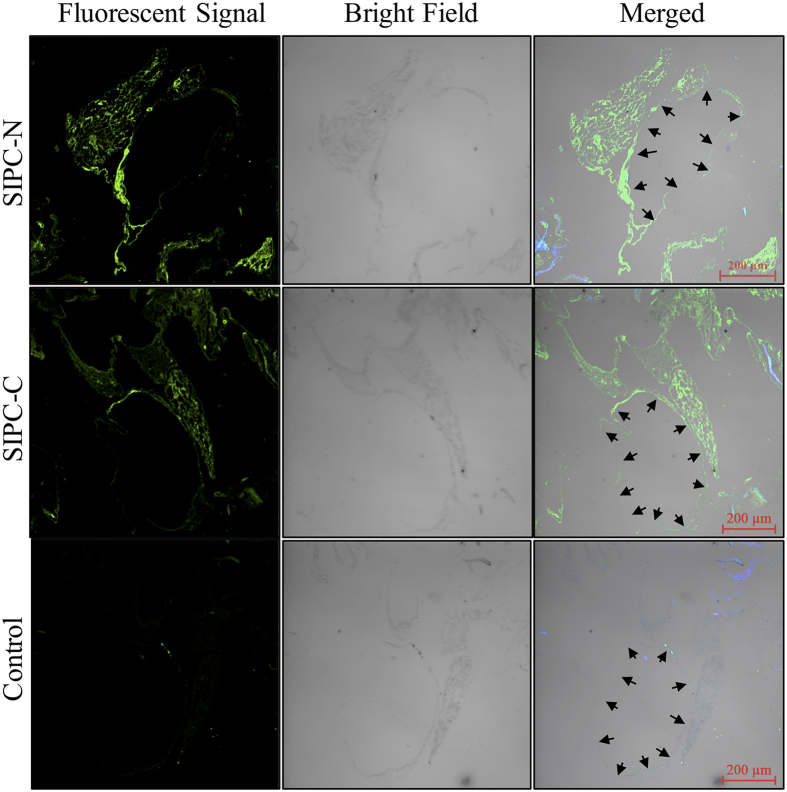
Fluorescent immunostaining for SIPC in decalcified barnacle shell sections. SIPC-N and SIPC-C represent antibodies against the N and C termini of SIPC, respectively. The black arrows indicate the contours of the lacunae (large channel) inside the shells. The 3D view of the FI results from the SIPC-N antibody was processed using IMARIS and is presented as Supplementary Movie S2. This experiment was repeated three times and one group of images were presented.

The FI results using antibodies against the N and C termini of SIPC both revealed strong signals in the residual matrix structures (Fig. 3). Regular large channels, or lacuna, are present inside the paries of the barnacle shell. SIPC was localized to the contours of the lacunae (Fig. 3). A Z-stack observation of the signals revealed that the SIPC-immunoreactive structure was a network (Supplementary Movie S2).

The decalcified shell sections were coated with gold and observed using a SEM. The results showed typical rhombohedral calcite crystals that were combined with the residual shell matrix (Supplementary Fig. S2). In the higher magnification images, the matrix appeared to be composed of fibers, and many granular structures were observed on the surfaces of these fibers (Supplementary Fig. S2).

The shell pieces (without decalcification) were analyzed by I-SEM. Energy-dispersive X-ray spectroscopy (EDXS) analysis revealed clear silver signals in these pieces (Supplementary Fig. S3). In the longitudinal sections isolated from the center of a lacuna, silver was localized to the calcareous paries. In the cross-sections at the bottoms of the shells adjacent to the basal plate, silver signals were localized to the calcified minerals, with relatively stronger signals detected at the contours of the lacunae and membranes attached to the channels (Supplementary Fig. S3).

### Recombinant expression of SIPC

Full-length SIPC was expressed using the baculovirus-insect cell expression system. The molecular weight of recombinant SIPC (recSIPC) was estimated to be 200 kDa using SDS-PAGE (Supplementary Fig. S4A). In a 5% native PAGE gel, recSIPC consisted of two bands at ~400 kDa and slightly less than 1,000 kDa (Supplementary Fig. S4B). These two bands were considered to be the dimer and tetramer of recSIPC. Western blot analysis revealed that heterogeneously expressed SIPC had a similar molecular weight to natural SIPC from barnacle extracts and Pro-Q 488 glycoprotein staining displayed a band of recSIPC in the PAGE gel (Supplementary Fig. S4C,D), which suggested that the recSIPC expressed using the baculovirus-insect cell expression system was post-translationally modified.

Interestingly, recSIPC could not induce barnacle larval settlement, when recSIPC was either added into seawater or absorbed on a PVDF membrane.

### recSIPC binds to chitin

**Figure 4:**
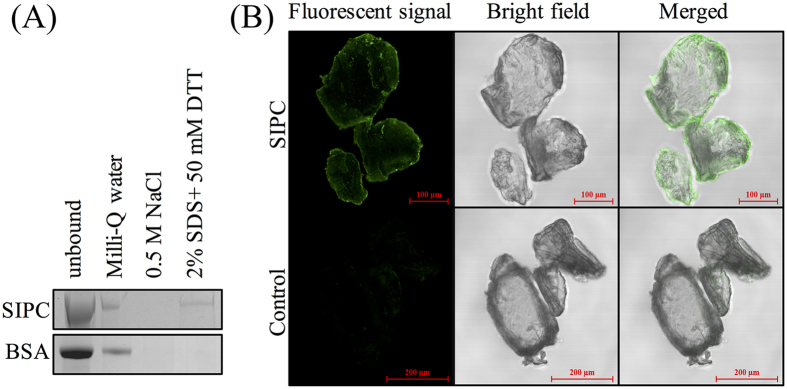
recSIPC binds to chitins. (**A**) recSIPC or BSA was incubated with chitin and then eluted using Milli-Q water, 0.5 M NaCl and a denaturation buffer (2% SDS + 50 mM DTT), sequentially. recSIPC was detected in the denaturation fraction but was absent in the NaCl fraction. (**B**) Chitins incubated with recSIPC solution were immunostained using a recSIPC antibody. The results strongly indicated recSIPC attachment to chitins. Chitins incubated directly with the recSIPC antibody and secondary antibody served as a control. These experiments were repeated three times and one group of images were presented.

After an overnight incubation with chitins, recSIPC was sequentially eluted using Milli-Q water, 0.5 M NaCl solution and a denaturation buffer (2% SDS + 50 mM DTT). SIPC was absent in the 0.5 M NaCl fraction but present in the denatured fraction (Fig. 4A). Chitins incubated with and without recSIPC solution were immunostained using recSIPC antibody. The results indicated that recSIPC was able to bind to the surfaces of chitin pieces (Fig. 4B).

### recSIPC induces CaCO_3_ precipitation

**Figure 5:**
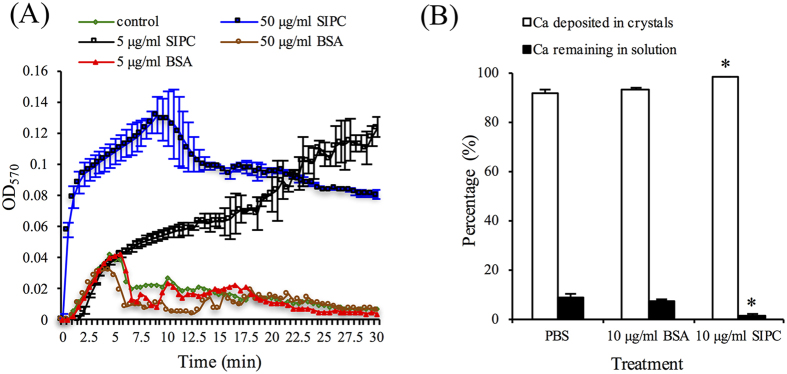
recSIPC induces deposition of CaCO_3_. (**A**) A mixed solution of CaCl_2_ and NaHCO_3_ was used to precipitate CaCO_3_. OD_570 nm_ represents the turbidity of the reaction system. The error bars in the BSA and control treatments overlapped with one other and thus were not labeled. (**B**) CaCO_3_ crystallization was performed with and without recSIPC/BSA. The ratio of Ca in the crystals and solution was determined. A greater amount of Ca was deposited into crystals treated with recSIPC compared with BSA treatments or the control (PBS alone). The data presented in the figure represent the mean ± SD from three replicates (n = 3).

A mixture of NaHCO_3_ and CaCl_2_ solution was used to precipitate CaCO_3_. There were no significant differences in the turbidity of the reaction systems between the 5 or 50 μg/ml BSA (in PBS) and the blank control (PBS alone) treatments. In contrast, both 50 and 5 μg/ml recSIPC rapidly increased the turbidity of the reaction system. The turbidity of the 50 μg/ml recSIPC system decreased gradually after 10 min, which was attributed to the aggregation and sedimentation of CaCO_3_ particles (Fig. 5A).

### recSIPC regulates CaCO_3_ crystallization

CaCO_3_ crystals were grown using (NH_4_)_2_CO_3_ and CaCl_2_ solution. Either 2 μg/ml recSIPC or BSA (dissolved in 10 μl PBS) or PBS alone was added to the reaction system. After 12 hours, the quantity of Ca in solution and crystal form was both determined. Approximately 91.3% and 92.9% of the Ca was deposited in crystals in the BSA and control treatments, respectively. In comparison, a significantly higher percentage of (98.4%) Ca was deposited in the recSIPC treatment (Student’s *t*-test, *p* < 0.05; Fig. 5B).

**Figure 6:**
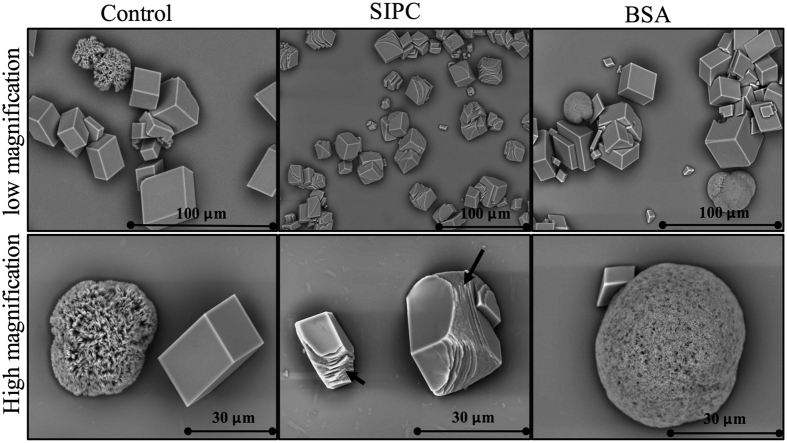
recSIPC affects CaCO_3_ crystallization. CaCO_3_ crystals were grown in 11 mM CaCl_2_ solution through the diffusion of CO_2_ from (NH_4_)_2_CO_3_. Two types of crystals, rhombohedral calcite and spherical vaterite, were obtained in the BSA and control (PBS) treatments. Only rhombohedral calcites were found in the recSIPC treatment. Layered structures at the edges of the calcite crystals were observed in response to the treatment with recSIPC and are indicated by black arrows. This experiment was repeated three times and one group of figures were presented.

The formed crystals were observed using a SEM. Two types of CaCO_3_ crystals, including typical rhombohedral calcite and spherical vaterite, were observed in both the BSA and control treatments, but only one type of crystal (rhombohedral calcite) was observed in the recSIPC treatment. The calcite crystals resulting from treatment with recSIPC were relatively smaller than the crystals from the BSA and control treatments. Morphologically, the calcite crystals generated from the BSA and control treatments were in the typical rhombohedral shape and exhibited smooth surfaces with rigid and sharp edges. However, the calcite crystals that were generated from treatment with recSIPC showed layered structures on their surfaces and curving edges (Fig. 6).

Immunostaining revealed a strong recSIPC signal in the crystals that formed with recSIPC (Supplementary Fig. S5), confirming that recSIPC participates in crystallization. However, the binding assay between recSIPC and CaCO_3_ crystals revealed that recSIPC bound to vaterites but not calcites (Supplementary Fig. S6). Crystals that were not pre-incubated with the recSIPC solution served as a control. Unexpectedly, the crystals in the control group dissolved after several washings during the experimental process. Ultimately, only some pieces of debris were retained, and no fluorescent signals were detected (Supplementary Fig. S6).

### Stability of the CaCO_3_ crystals

**Figure 7:**
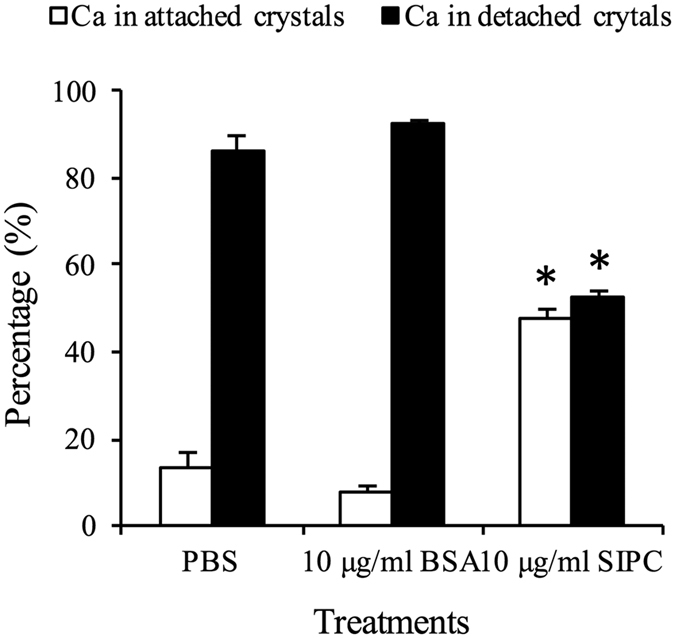
Stability of CaCO_3_ crystals. Crystals formed on coverslips with BSA/recSIPC or PBS alone were soaked in Milli-Q water for 60 hours. The Ca distribution between detached (Milli-Q water) and attached crystals was determined and calculated. Significantly less Ca was dissolved in Milli-Q water or detached from the coverslips in the recSIPC treatment compared with the BSA treatment and the control. No significant differences were detected between the BSA treatment and the control. The data presented in the figure represent the mean ± SD from four replicates (n = 3).

Crystals that had formed on coverslips under different conditions were soaked in Milli-Q water for 60 hours. Most of the crystals that formed in the BSA or control treatments detached from the surfaces of the coverslips. These crystals moved along with a water current generated by a pipette. In comparison, the crystals that formed with recSIPC still showed a high binding affinity to the surface of the coverslips. The crystals remained attached when the water current hit them (Supplementary Movie S3). The ratio of detached to attached crystals was determined using an inductively coupled plasma optical emission spectrometer (ICP-OES). Approximately 90% of the Ca detached in the BSA and control treatments. In comparison, only 52% of the Ca detached in the recSIPC treatment (Fig. 7). Scanning electron microscopy showed that the detached crystals were seriously damaged and partially dissolved. The surfaces of the attached crystals were etched and became unsmooth (Supplementary Fig. S7).

## Discussion

Dreanno *et al*.^6^ hypothesized that SIPC was released into seawater from cuticles through bacterial digestion during molting or cuticle regeneration^6^. In the present study, I-SEM images directly revealed positive SIPC signals in both carapace pores and frontal horn pores. The carapace pores are the external openings of the paired epidermal glands^26^,^27^. Similarly, a pair of larger, histologically similar epidermal glands are situated underneath the frontal horn pores^26^. The epidermal glands can secrete a wetting and proteinaceous agent^28^,^29^. In barnacle nauplii, nerves were observed underneath the frontal horn glands, suggesting these glands might have sensory capacity^30^. The present results suggest another possibility: that is, SIPC is actively released from carapace pores and frontal horn pores in *A. amphitrite* cyprids.

While exploring their environments, cyprids “walk” on surfaces and leave deposits on the surfaces. During this process, the ventral area corresponding to the frontal horn pores touched the surface frequently while the cyprids were walking (Supplementary Fig. S8 and Supplementary Movie S4), which was also reported by Lagersson and Høeg^31^. Further immunostaining for SIPC on the cyprid-interacted surfaces displayed positives signals of SIPC. These signals were hill-like when viewed in 3D and crescent or triangular in shape. At some extent, the shape of these signals was similar to the frontal horn pores. The length of the SIPC-immunoreactive deposits (5–10 μm) was also similar to the length of the frontal horn pores (8.8–16.7 μm) (the present study and^26^). These results raised a possibility that frontal horn pores might deposit SIPC to the touched surfaces during “walking”.

Previously, SIPC in barnacle shells was thought to function as a contact pheromone for conspecific gregarious settlement^6^. In the present study, SIPC bound to a fiber network primarily composed of chitin in barnacle shells. This network not only combined with calcite crystals but also exhibited a morphology similar to the shell matrix of the mollusk *Atrina rigida* (globular structures distributed along fibers)^32^. Additional binding and immunostaining results showed that recSIPC could tightly bind to chitins *in vitro*. Taken together, these results suggest that SIPC binds to chitin. Previously, we found that SIPC could be extracted from barnacle shells only in the presence of SDS and DTT^16^, which might be due to the tight binding between SIPC and chitins in barnacle shells.

In the natural environment, CaCO_3_ forms three anhydrous crystalline polymorphs: calcite, aragonite and vaterite^33^. Vaterite was not observed in the recSIPC treatment in the present study, which suggested that recSIPC might inhibit the formation of vaterite. This hypothesis was also supported by the binding affinity observed between recSIPC and vaterites. In general, when specific proteins in solution interact with a previously formed crystal, crystal growth is inhibited^34^. Calcite is the only CaCO_3_ polymorph found in *A. amphitrite* shell minerals^20^,^35^, a phenomenon that might be due to the presence of SIPC in barnacle shells. Currently, the mechanism by which recSIPC inhibits vaterite formation still remains unknown. Nevertheless, it is generally accepted that crystal growth is inhibited by the interaction between the growth sites of crystals and the carbonyl oxygen atoms in the side chains of acidic amino acid residues and their amide forms^36^. Furthermore, only rhombohedra with very smooth surfaces was observed in the BSA and the control treatments. However, the crystals grown with recSIPC displayed macrostepping at the crystal edges. This special morphology is similar to that observed for crystals formed with EDTA-soluble proteins extracted from the shell of *Mytilus edulis*^37^ or AP8 proteins, which are extremely acidic proteins that are purified from abalone nacres^38^. Thus, recSIPC might also function in the biomineralization process. The function of recSIPC in biomineralization might be related to its sugar chains, because treatment with polysaccharides from intracrystalline glycoproteins similarly resulted in crystals with rough curved surfaces capped by rhombohedral faces^19^. Mannose and glucose from bacterial exopolysaccharide, which are also the main components of SIPC sugar chains^25^, have been approved to regulate the morphology of calcite crystals^14^. It would be better to test the effects of deglycosylated SIPC on crystal formation. However, it is very difficult to remove SIPC sugar chains and subsequently recover the protein backbone under native conditions.

Although the physiochemical parameters (amino acid composition, pI, molecular weight and post-translational modifications, and others) of recSIPC are completely different from AP8 proteins, both of these molecules might function as nucleators in the biomineralization process. The immunostaining results demonstrated the presence of recSIPC in calcite crystals grown with recSIPC, but the binding assay did not reveal strong signals indicative of the attachment of recSIPC to the surface of calcite crystals. These results indicated that recSIPC might be deposited within calcite crystals rather than being attached to the crystal surfaces, suggesting that recSIPC plays a role as a nucleator. The CaCO_3_ precipitation assay involved the following chemical reactions: Ca(HCO_3_)_2_ = CaCO_3_ + CO_2_ + H_2_O. recSIPC accelerated and increased the precipitation of CaCO_3_, suggesting that SIPC promoted this reaction to precipitate more CaCO_3_. The most likely mechanism underlying this process is recSIPC-triggered nucleation of CaCO_3_. During the growth of the treated crystals, a greater number of smaller calcite crystals were obtained with recSIPC compared with the crystals obtained from the BSA and control treatments, further suggesting that recSIPC might function as a nucleator. Overall, these results support the hypothesis that recSIPC might serve as a nucleator during the crystallization of CaCO_3_.

In many mineralized tissues, there is a distinction between matrix macromolecules located outside the crystals (intercrystalline) and the molecules located within the crystals (intracrystalline)^19^. The present study revealed that recSIPC exhibited both chitin binding (intercrystalline) and crystal nucleation (intracrystalline) properties. This finding is not an isolated event; other glycoproteins have also been identified as both intercrystalline and intracrystalline macromolecules^19^. The dual roles of recSIPC might have a special biological significance to barnacles. Nucleation occurs when specific proteins are adsorbed onto a rigid substrate^34^. The CaCO_3_ crystal growth assay revealed that calcite crystals could form on chitin surfaces with or without recSIPC (Supplementary Fig. S9). In barnacle shells, the binding of SIPC and chitins might create a local supersaturation of CaCO_3_ to enable the deposition of CaCO_3_ crystals around the chitin framework. The calcite crystals that formed with recSIPC displayed an increased stability that might contribute to the rigidity of the barnacle shells. A thin layer of membrane surrounds the surfaces of channels inside barnacle shell paries, a location characterized by shell growth^39^ and the presence of epithelial cells^40^. SIPC, newly formed crystals and chitin network-like structures were observed in this membrane by immunostaining, SEM and HE staining, respectively (Supplementary Fig. S10). The co-localization of these three elements suggested that SIPC might regulate shell formation *in vivo*.

## Conclusions

In barnacle cyprids, SIPC can be released from frontal horn pores and carapace pores. When cyprids explore their environments, frontal horn pores could touch surfaces and might deposit SIPC onto the surface. In barnacle shells, SIPC might participate in the biomineralization process, through accelerating the deposition of minerals around a chitin framework and stabilizing the binding strength between calcite and chitin.

## Methods

### Ethics statement

No specific permit is needed for barnacle studies in Hong Kong. During this study, all experiments were performed in accordance with relevant guidelines and regulations. The location at which the barnacles were collected does not belong to any national parks, protected areas or private lands. There were no protected species in the sampling area, and no local laws or regulations were overlooked.

The protocols for the production of antibodies against SIPC using rabbits were approved by the Department of Health, the Government of the Hong Kong Special Administrative Region (Ref. no: (14–39) in DH/HA&P/8/2/2 Pt.6) and the Animal Ethics Committee at the Hong Kong University of Science and Technology (Ref. no: 2014042). The methods were carried out in accordance with the approved guidelines.

### Barnacle sample preparation

To collect barnacles for studies conducted in our laboratory, PVC plates (20 × 30 cm) are routinely hung at the intertidal area at Tso Wo Hang Pier in Sai Kung, Hong Kong (22°23′31.30“N, 114°17′18.34”E) for natural colonization by *A. amphitrite*. Barnacle larvae were released and cultured as described previously^41^. Cyprids were relaxed in 0.37 M MgCl_2_ for 30 min and then fixed in 4% paraformaldehyde (PFA) in PBS at 4 °C overnight.

Glass coverslips (25 × 25 mm) were coated with 0.05% poly-L-lysine in Milli-Q water for 2 hours. Next, the coverslips were washed 3 times with 0.22-μm-membrane-filtered seawater (FSW) and placed in 6-well tissue culture plates. Approximately 20 cyprids were transferred into each well and incubated for 5 hours. During this time, the cyprids left deposits on the coverslips. After washing 3 times with FSW, the coverslips were immersed in 4% PFA for fixation.

The shells were separated, cleaned carefully with FSW and fixed in 4% PFA. Next, the shells were decalcified in a decalcification solution (5% hydrochloric acid, 7% formic acid, 5% CaCl_2_, 2.5% acetic acid and 10% formaldehyde in PBS) for 20 min. The residual shell matrix was washed three times with PBS.

Histological sections of the cyprids and the shell matrix were obtained. After washing with PBS for 15 min × 3 times to remove the PFA, the samples were dehydrated in ethanol, infiltrated with xylene and finally embedded in paraffin according to an established protocol^42^. Both the shell matrix and cyprids were cut into 4-μm-thick layers, dewaxed in xylene, rehydrated in ethanol solutions and washed with PBS before immunostaining.

### SIPC antibody generation in rabbits

Total RNA extraction from adult *A. amphitrite* barnacles, cDNA synthesis and purification were conducted as described by Zhang *et al*.^41^. Two segments, one spanning amino acids 18–117 (N termini) and the other spanning amino acids 1318–1429 (C termini), were cloned. These two segments were both expressed as fusion proteins with His_6_ (for antigen injection) and the GST tag (for antibody purification). The protocol for antigen injection and antibody purification has been described previously^41^.

The Western blot and immunoprecipitation analysis results suggest that our antibodies are effective and specific to SIPC (Supplementary Fig. S11).

### Fluorescent immunostaining

Immunostaining was performed using cyprid and shell matrix sections, whole mounts of cyprids and shell pieces. The samples were washed with PBS to remove the fixatives. The sections were heated at 95 °C in a water bath for 10 min. Next, all of the samples were treated with 1% triton X-100 for 30 min and blocked in 3% BSA in PBS for 2 hours at room temperature. The samples were subsequently incubated with primary polyclonal antibodies at 4 °C overnight, washed 3 times with PBS, incubated with a goat anti-rabbit IgG secondary antibody (labeled with 10 nm colloidal gold or Alexa Fluor 488 conjugate; Life Technologies, Prakley, UK). After washing 3 times with PBS, the samples were mounted and observed under a Zeiss laser scanning confocal microscope (LSM710 DUO, Zeiss). The confocal microscopy data were further processed using IMARIS software (Bitplane Company, Zurich, Switzerland).

### Immuno-scanning electron microscopy

The immunostaining procedure for sections and whole mounts of cyprids and shell pieces for I-SEM observation were conducted as described above. The secondary antibody was labeled with 10 nm colloidal gold particles. It was difficult to observe these tiny particles under SEM. For easy observation, the samples were washed 3 times with Milli-Q water and developed using a LI silver (LIS) enhancement kit (Life Technologies, Prakley, UK) for 10 min. Silver was deposited onto the colloidal gold particles, enlarging them to 30–100 nm in diameter, which then could be observed using SEM. The samples were then fixed in 2.5% glutaraldehyde for 30 min to stabilize the antibody-antigen interactions. The samples were washed with Milli-Q water, dehydrated in an ethanol gradient, transferred into t-butanol for 3 rinses, and dried using a freeze drying device (VFD-21S, Vacuum Device Co. Ltd., Mito, Japan).

The dried samples were observed directly (without any coating) under a TM3030 tabletop SEM (Hitachi, Japan) coupled with an EDXS system. Under the charge-reduced model, the distribution of silver was recorded using the EDXS system. Alternatively, the samples were coated with carbon or gold and observed using a JSM 6390 SEM (JEOL, Peabody, MA, USA) under the secondary electron image (SEI) or backscattered electron composition image (BEC) models. The silver particles clearly appeared as white spots in the BEC model.

### Recombinant expression of SIPC in insect cells

To obtain full-length SIPC, SIPC was divided into two segments, and nested PCR was performed to amplify the two segments using the PrimeSTAR HS amplification premix (Takara, Japan). The two segments were validated using the Sanger sequencing service (BGI, Shenzhen, China) and combined using overlap PCR. The full-length SIPC cloned in this study was deposited in GenBank (accession no. KT630650). The primers used in this study are listed in Supplementary Table S1.

recSIPC was expressed in insect cells using a Bac-to-Bac^®^ Baculovirus Expression System (Life Technologies, Grand Island, NY, USA). Briefly, full-length SIPC was cloned into a pFastBac^TM^HT A vector (Life Technology, Grand Island, NY, USA) at both *Bam*H I and *Hin*d III sites using a CloneEZ PCR Cloning kit (Genscript, Nanjing, China) and transformed into Top10 competent *E. coli* cells. After verification using Sanger sequencing, the plasmid was extracted and then further transformed into DH10Bac^TM^ competent *E. coli* cells containing a baculovirus shuttle vector (bacmid). In the DH10 cells, SIPC sequence was transposited from the pFastBac^TM^HT A plasmid to the bacmid. Recombinant bacmids in DH10 colonies were verified using PCR and then extracted using a NucleoBond PC 20 Plasmid DNA purification kit (Macherey-Nagel GmbH & Co. KG, Düren, Germany). Next, 1 μg of purified recombinant bacmid DNA was transfected into 8 × 10^5^ Sf9 insect cells (Life Technology, Grand Island, NY, USA) in a 6-well plate using Cellfectin^®^ II reagent (Life Technology, Grand Island, NY, USA). After 3 days, the supernatants containing baculovirus were collected, which represented the P1 viral stock. Next, the P1 viral stock was amplified in healthy Sf9 insect cells to generate the P2 viral stock with a higher titer. The P2 viral stock was used to infect Sf9 insect cells in 300 ml of culture medium. After 3 days, the Sf9 insect cells were harvested and recombinant SIPC (His_6_ tagged) was purified using the Ni-NTA agarose kit (Qiagen, Santa Clarita, CA, USA) under denaturation conditions, dialyzed against descending concentrations of urea in PBS buffer at 4 °C to renature SIPC and concentrated using an Amicon Ultra centrifugal filter device (100 kDa cut-off, Millipore, Carrigtwohill, Ireland).

### Chitin binding assay

The chitin binding assay was conducted according to the method described by Folders *et al*.^43^ with some modifications. First, chitin (Sigma-Aldrich, St Louis, MO, USA) was washed with 0.2 M HCl and 0.2 M NaOH to remove any contaminating proteins and equilibrated with Milli-Q water. Next, 20 μg recSIPC and 2.5 mg chitin were incubated in 400 μl NH_4_HCO_3_ (25 mM) solution at 4 °C overnight. After centrifugation at 10 kg for 5 min at 4 °C, the supernatants were collected as the unbound recSIPC fraction, and the chitin pellets were washed 3 times with Milli-Q water, followed by 3 washes with 0.5 M NaCl solution (NaCl fraction). The 3 washes using water or NaCl solution were pooled and concentrated using Amicon Ultra centrifugal filter devices. As a last step, the chitin pellets were boiled in a denaturation solution (2% SDS and 50 mM DTT in 0.5 M NaCl solution) for 10 min. The proteins in each fraction were separated in a 4–20% gradient SDS-PAGE gel (Genscript, Nanjing, China) and stained using Coomassie blue G250. The same amount of BSA was used in place of recSIPC as a control.

To visualize the binding of recSIPC and chitin, chitin was blocked in 3% BSA in PBS at room temperature for 2 hours and incubated with 50 μg/ml recSIPC in blocking solution at 4 °C overnight. The chitin was washed with PBS for 3 × 15 min, incubated with recSIPC antibody at 4 °C overnight, washed 3 times with PBS for 15 min and incubated with an Alexa Fluor 488-labeled secondary antibody (Life Technologies, Carlsbad, CA, USA) at room temperature for 2 hours. The chitin was then washed with PBS and observed using a confocal microscope. Chitin incubated with BSA solution instead of recSIPC solution served as a control.

### CaCO_3_ precipitation test

The protocol for the CaCO_3_ precipitation test was modified from Inoue *et al*.^44^. Three hundred and ninety microliters of 66 mM NaHCO_3_ (pH 8.7) and 10 μl sample solution (4 or 40 μg recSIPC in PBS, 4 or 40 μg BSA in PBS, or PBS alone) were mixed in a 1-cm plastic cuvette. Following the addition of 400 μl of 66 mM CaCl_2_ solution to the cuvette, the turbidity of the solution was recorded every 30 sec for 30 min by monitoring the absorbance at 570 nm using a Beckman DU650 spectrophotometer (Beckman, Urbana, IL, USA). The absorbance represents the amount of CaCO_3_ precipitation. The experiments were repeated 3 times.

### CaCO_3_ crystal growth

CaCO_3_ crystallization was performed in 6-well polystyrene plates at room temperature using the gas diffusion method^45^. A piece of coverslip (22 × 22 mm) was soaked in 4 ml of 11 mM CaCl_2_ solution in each well. Next, 10 μl of sample solution (40 μg recSIPC or BSA in PBS or PBS alone) was added to each well. The plates and an open flask of (NH_4_)_2_CO_3_ powder were sealed in a desiccator containing dried silica gel blue. Ammonia carbonate powder automatically released CO_2_, which diffused into CaCl_2_ solution and formed CaCO_3_ crystals. After 12 hours, the solutions were collected, and the crystals were washed 3 times with Milli-Q water. All of the solutions were pooled and acidified to a pH < 2 (7% HNO_3_). The crystals were dissolved in 7% HNO_3_. Next, the concentration of Ca in these two fractions was determined using an ICP-OES (725-ES, Varian, Australia) with 3 technical repeats. The percentage of Ca in the crystals and solutions was calculated. This assay was repeated 3 times (experimental replicates), and the results from different treatments and the control were compared using a Student’s *t*-test and SPSS 11.5 software.

Another batch of crystals was grown as described above. The crystals grown on coverslips were observed under an optical microscope and then washed with Milli-Q water, dehydrated with an ethanol gradient, air-dried and observed under a SEM (TEM3030, Hitachi, Japan) in the charge-reduced model.

The crystals grown with recSIPC were immunostained to investigate the deposition of recSIPC in the crystals. A SIPC-CaCO_3_ crystal binding assay was performed using the same protocol described in the chitin-binding assay. Crystals treated with the secondary antibody alone served as a control.

### Stability of the CaCO_3_ crystals

Crystals grown on coverslips in the presence of 10 μg/ml BSA/recSIPC in PBS or PBS alone were soaked in 5 ml of Milli-Q water with gentle shaking. The Milli-Q water was changed every 12 hours for a total of 5 changes. For each sample, all of the collected Milli-Q water was pooled and adjusted to 30 ml and a pH < 2. The crystals that were still attached to the coverslips were dissolved in 7% HNO_3_, and the volume was adjusted to 10 ml. The concentration of Ca in these solutions was determined using ICP-OES, and the ratio of Ca attached to/detached from the coverslips was calculated. The experiments were repeated 4 times, and the means of the different treatments were compared using a Student’s *t*-test with SPSS 11.5 software.

## Additional Information

**How to cite this article**: Zhang, G. *et al*. Secretory locations of SIPC in *Amphibalanus amphitrite* cyprids and a novel function of SIPC in biomineralization. *Sci. Rep.* **6**, 29376; doi: 10.1038/srep29376 (2016).

## Supplementary Material

Supplementary Movie S1

Supplementary Movie S2

Supplementary Movie S3

Supplementary Movie S4

Supplementary Information
