# Supplementary material for: Secretory locations of SIPC in Amphibalanus amphitrite cyprids and a novel function of SIPC in biomineralization
Source: Sci Rep. 2016 Jul 20;6:29376. doi: 10.1038/srep29376 (PMC4951644; doi:10.1038/srep29376)
Supplement: Supplementary Information [file srep29376-s5.pdf]

Secretory locations of SIPC in *Amphibalanus amphitrite* cyprids and a novel function of SIPC in biomineralization

Gen Zhang[1], Xiao-Xue Yang[1], Pok Man Leung[1], Li-Sheng He[2], Tat Yin Chan[1], Guo-Yong Yan[2], Yu Zhang[3], Jin Sun[1], Ying Xu[3] & Pei-Yuan Qian[1*]

Supplementary Table S1. Primers used in this study.

Name	Sequence	Purpose
SIPC-N1-F	GAAGAGGACATGGGTGGTCC	1st round of nested PCR for N terminal of SIPC
SIPC-N1-R	GCTTCATCGAGTACGGCAGA	1st round of nested PCR for N terminal of SIPC
SIPC-N2-F	TCGTTCTACTGGTCGCCTTG	2nd round of nested PCR for N terminal of SIPC
SIPC-N2-R	GACAGTCTTCACACCCTCCG	2nd round of nested PCR for N terminal of SIPC
SIPC-C1-F	GCTTACTGGTCAGTCCACCC	1st round of nested PCR for C terminal of SIPC
SIPC-C1-R	AGAACTGTGATTCGCTGCCT	1st round of nested PCR for C terminal of SIPC
SIPC-C2-F	AGACACTAGATGCGGAGGGT	2nd round of nested PCR for C terminal of SIPC
SIPC-C2-R	TATGTTCCGCTTGGGCCTTC	2nd round of nested PCR for C terminal of SIPC
SIPC-N-anti-F-BamHI	TTTTggatccGTCAAGGTCCCCGAAAGCGG	antigen expression of N terminal of SIPC
SIPC-N-anti-R-XhoI	TTTTctcgagTCAGCTCCATGTGCATGTCAG	antigen expression of N terminal of SIPC
SIPC-C-anti-F-BamHI	TTTTggatccGCCATTCGCTGGATCAACAC	antigen expression of C terminal of SIPC
SIPC-C-anti-R-NotI	TTTTgcggccgcCTA CACACCGAGAGAGAAGGCAG	antigen expression of C terminal of SIPC
SIPC-F-BamHI-EZ	TGTATTTTCAGGGCGCCATGggatccGTCAAGGTCCCCGAAAGCGG	cloning of total length of SIPC into pFastBac HT A vector
SIPC-R-HindIII-EZ	TCCTCTAGTACTTCTCGACaagcttCTAAGCAGCGGGAGTCAGCT	cloning of total length of SIPC into pFastBac HT A vector
SIPC-F172	CTGTTCAACCTACCCGGACC	Sequencing
SIPC-R1520	GGAGTGCGAGCTGTCAGAAT	Sequencing
SIPC-F1465	CAGGCTGAGGACATCGACTC	Sequencing
SIPC-R2731	GGCAGAGACACTTCGGTGAA	Sequencing
SIPC-F2585	AGACACTAGATGCGGAGGGT	Sequencing
SIPC-R3929	CAGCGAGAGAGCCATGTAGG	Sequencing
SIPC-F3787	ATGGGCCAAGATGTCAAGGG	Sequencing
SIPC-R5026	CTTGCACAAACGATGCACCA	Sequencing

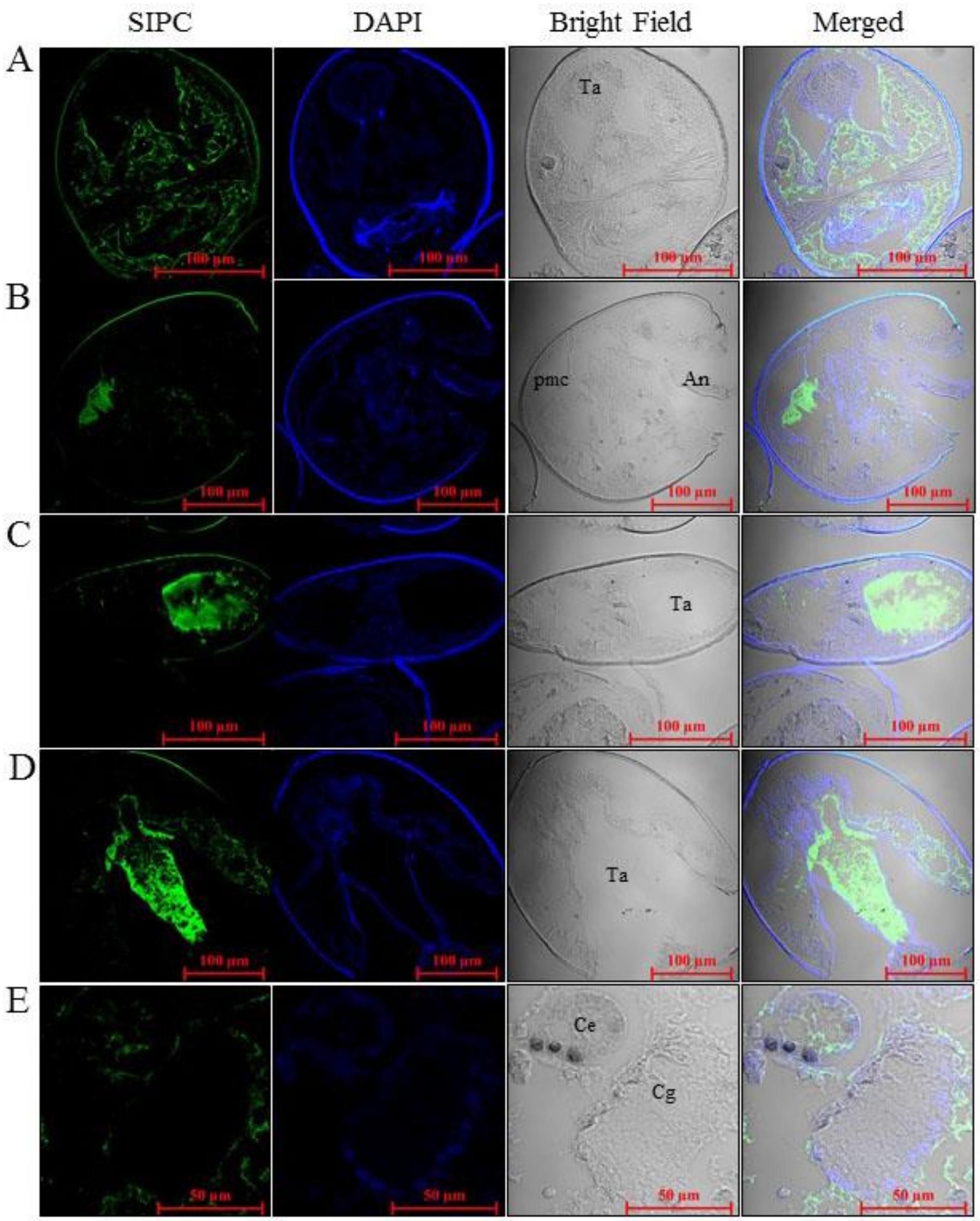

Supplementary Figure S1. Fluorescent immunostaining of SIPC in cyprids. (A). In the cross-sections, SIPC was localized to tissue spaces. (B-D). Strong SIPC signals were observed in the thoraco-abdomen. (E). SIPC signals were localized to the surface of the cement glands but not inside the cement glands. Ce=compound eye; Cg=cement gland; pmc=posterior mantle

cavity; Ta=thoraco-abdomen.

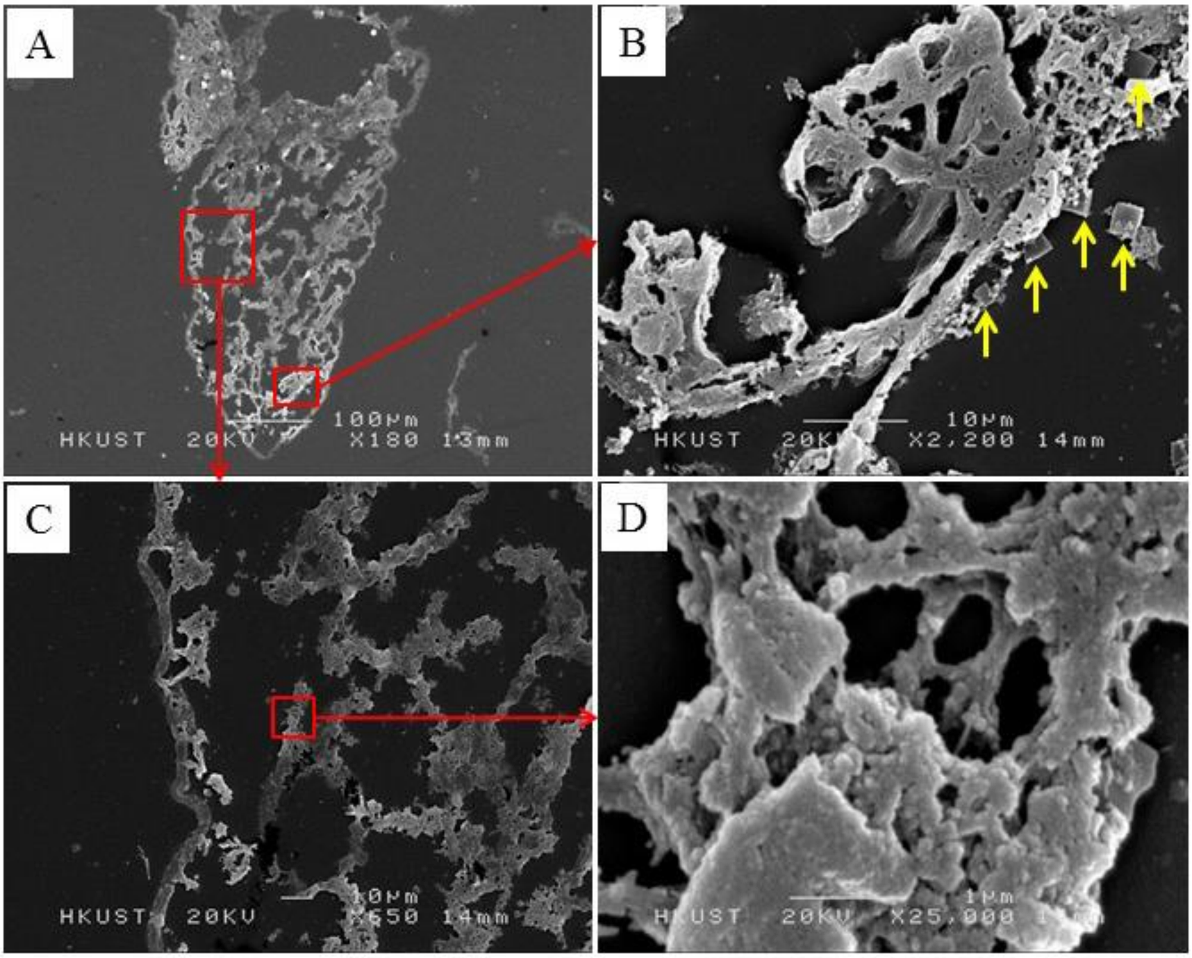

Supplementary Figure S2. SEM of decalcified shell matrix sections. (B)-(C): Higher magnifications of (A) at different areas. (D): A higher magnification of (C), in which many granular particles were observed on the surface of the fiber network. Yellow arrows indicate typical rhombohedral calcite crystals.

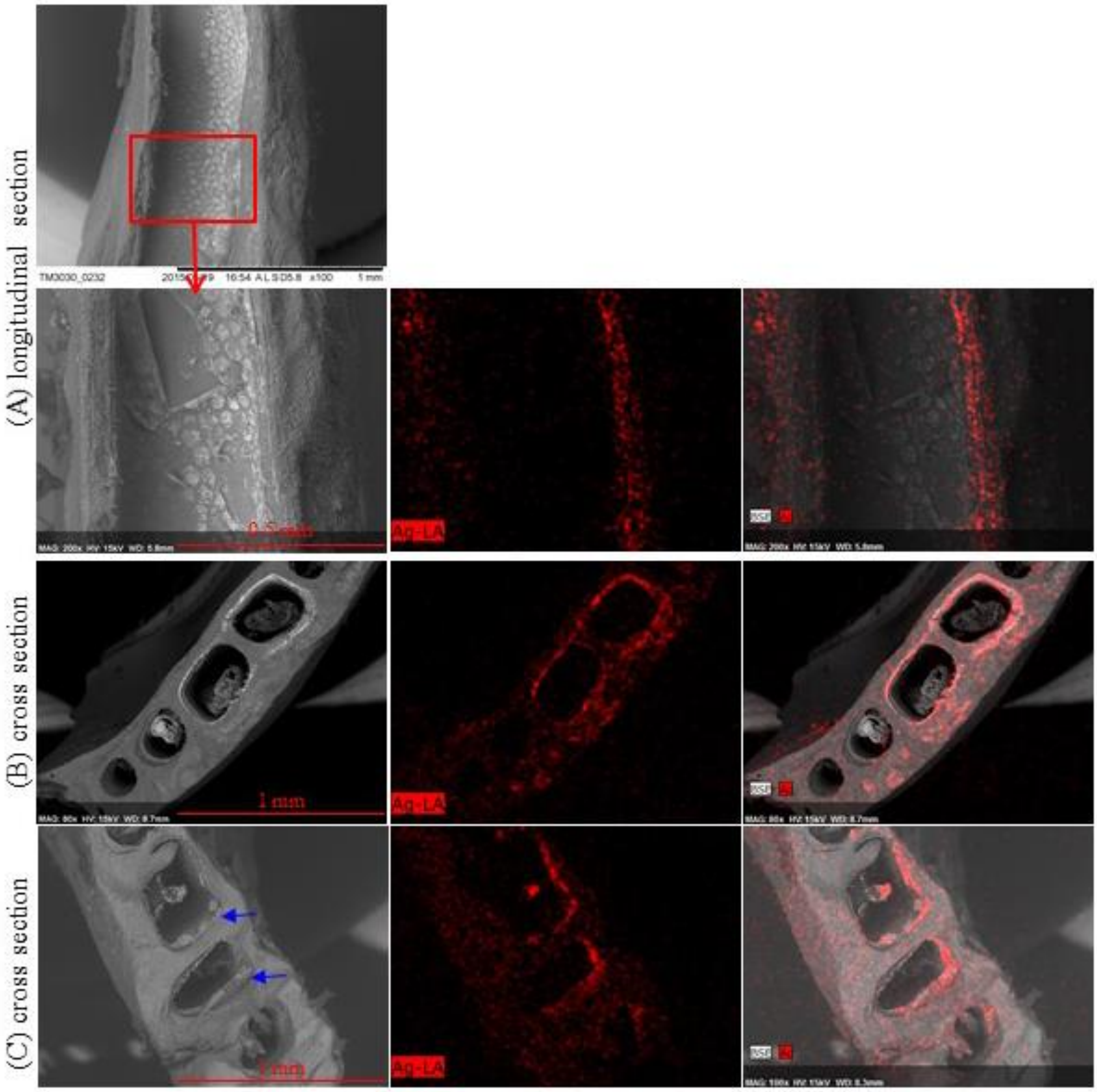

Supplementary Figure S3. I-SEM of SIPC on pieces of native barnacle shells. The red signals represent the distribution of silver using EDXS analysis. (A)-(B). SIPC signals were localized to minerals in barnacle shells. (C). A cross-section of a barnacle shell displaying a SIPC signal on the membrane attached to the surface of inner channels, which are indicated by blue arrows.

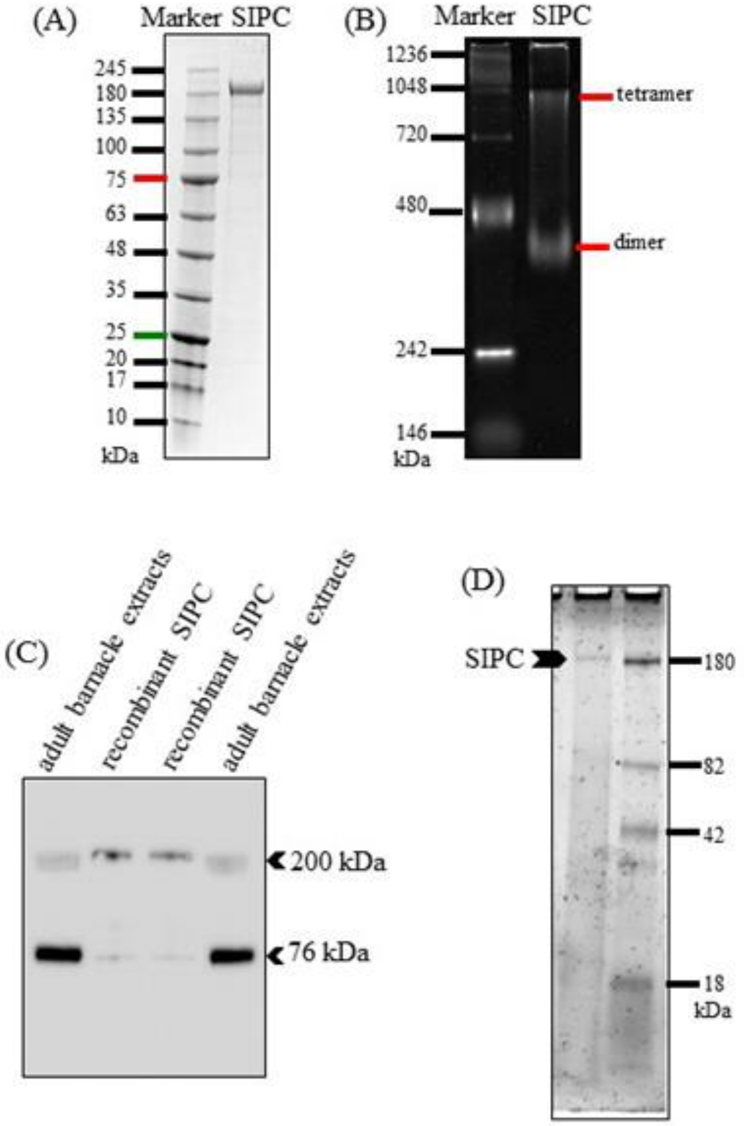

Supplementary Figure S4. Recombinant expression of SIPC. (A). recSIPC showed a single band on a 4-20% gradient SDS-PAGE gel that was stained with Commassie blue. (B) recSIPC displayed 2 bands, ~400 kDa and slightly below 1,000 kDa, on a 5% native PAGE gel. The gel was stained using SYPRO Ruby (Life Technologies, Grand Island, NY, USA). (C) Western blot analysis using an antibody against the C-terminus of SIPC detected 2 bands in adult barnacle extracts that corresponded to ~200 and 76 kDa. Similarly, the recSIPC expressed in insect cells also revealed 2 bands by Western blotting. One was slightly larger than the natural SIPC, potentially due to the extra sequence from the expression vector. The other band was equivalent

in size to the 76-kDa subunit of natural SIPC. (D) recSIPC was separated in an 8% PAGE gel and then stained using the Pro-Q® emerald 488 glycoprotein gel and blot stain kit (Life Technologies, Grand Island, NY, USA). A clear band of recSIPC was displayed by the glycoprotein staining. The CandyCane™ glycoprotein molecular weight standards (Invitrogen, Cat. No. C21852) were used as both positive and negative controls. Four bands in the standards were glycosylated and positively stained in present study.

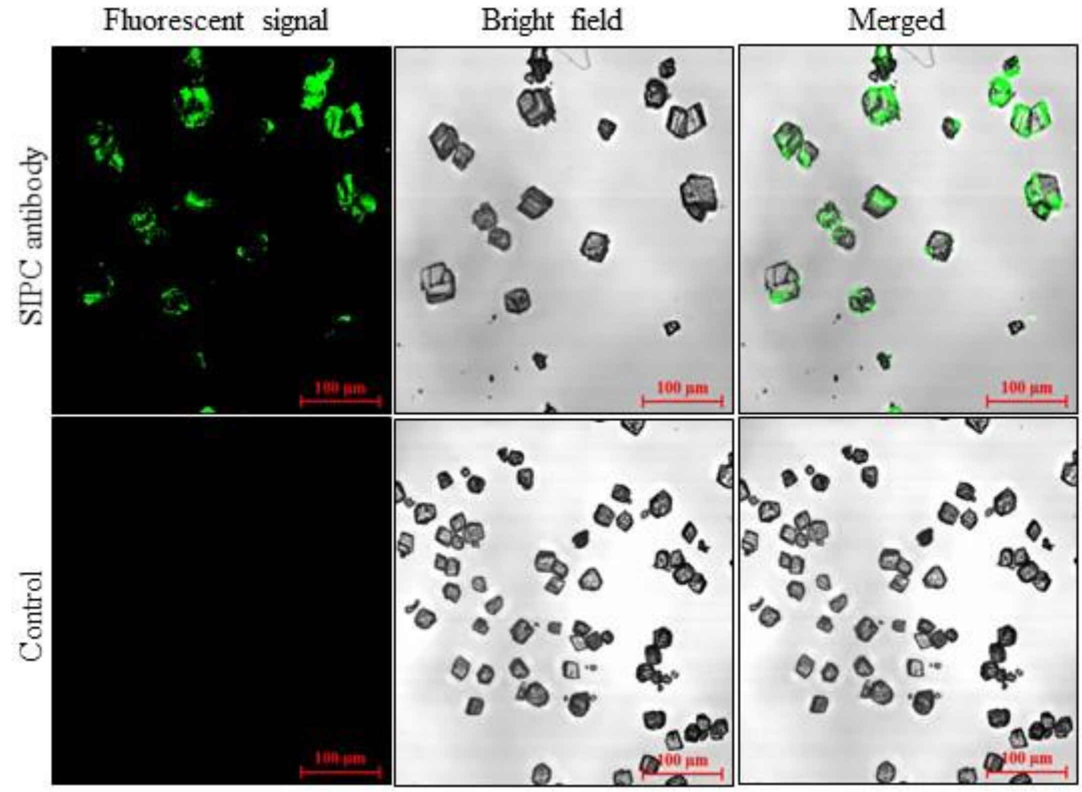

Supplementary Figure S5. Crystals grown with recSIPC were immunostained using a SIPC antibody. Rhombohedral calcites displayed positive signals.

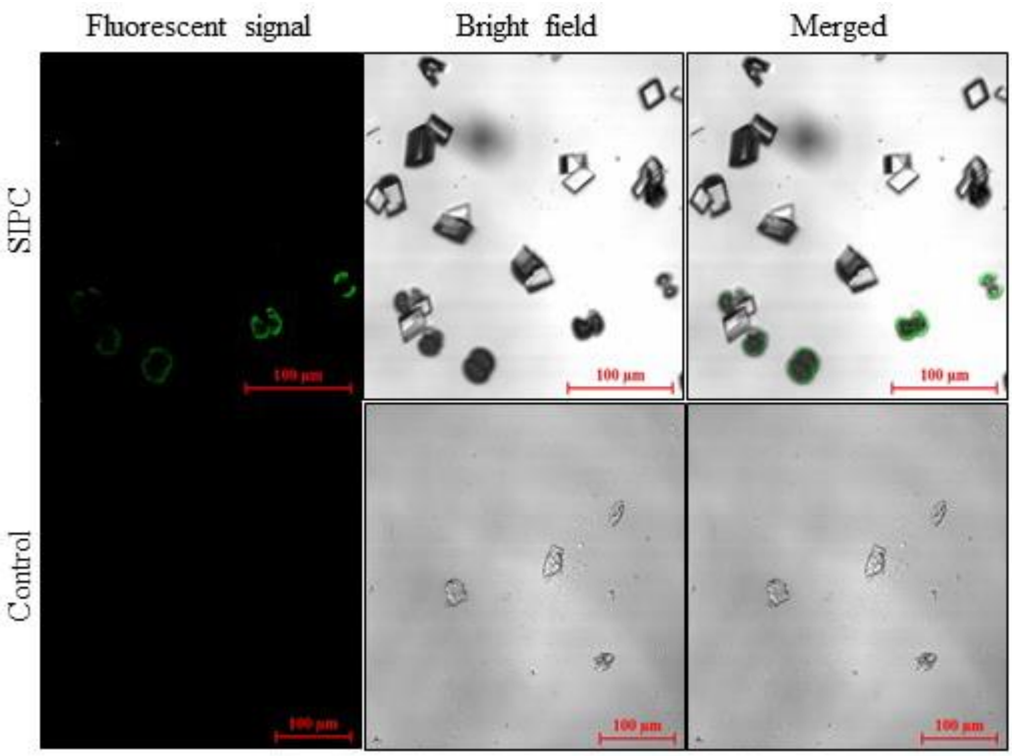

Supplementary Figure S6. recSIPC binds to vaterites but not calcites. Crystals grown without BSA or recSIPC were incubated with a recSIPC solution and then immunostained using an antibody against SIPC. Strong recSIPC signals were detected on vaterites but not calcites. In the control, crystals were directly immunostained with the SIPC antibody without previous incubation with a recSIPC solution. However, these crystals dissolved during the experimental process. The residual debris did not show any positive signals.

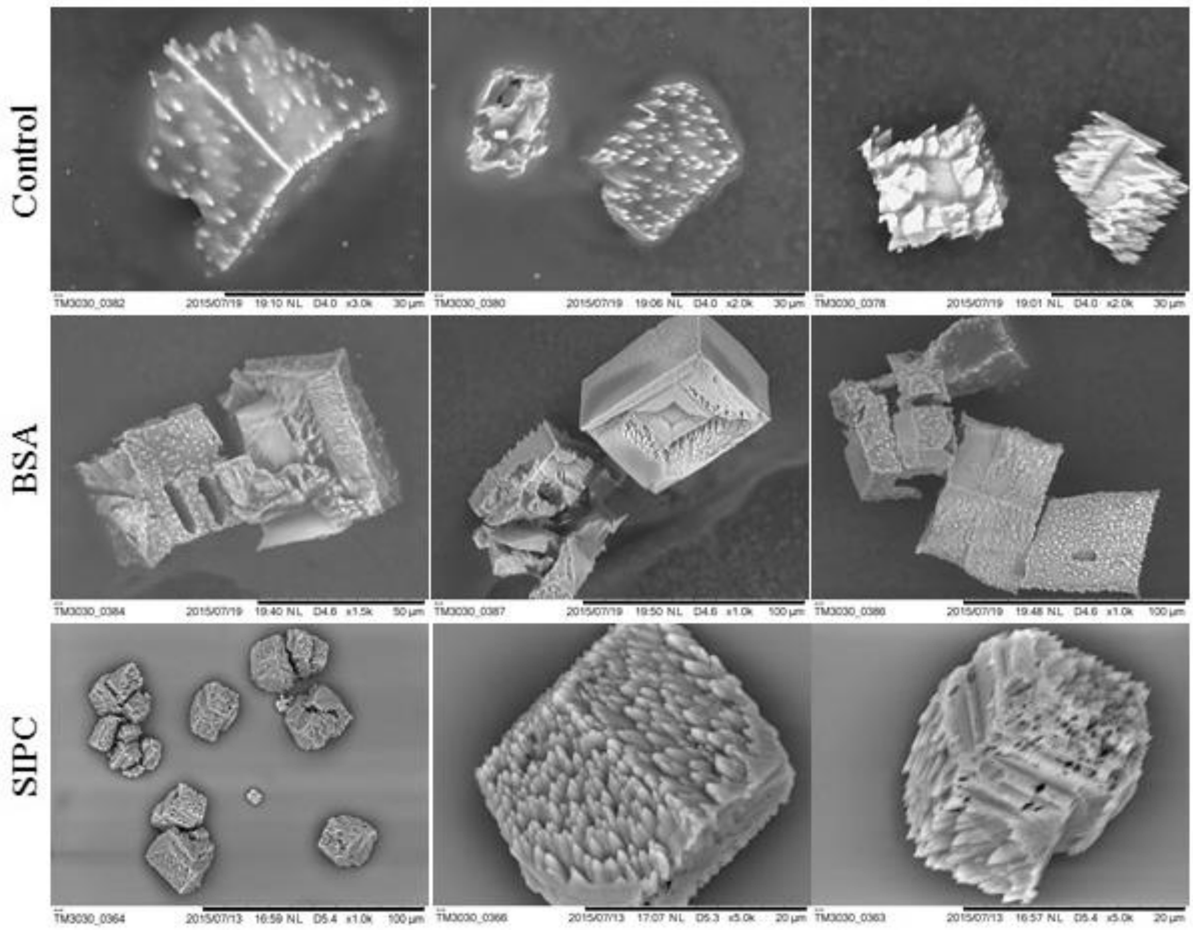

Supplementary Figure S7. Morphological changes in crystals formed under different conditions after soaking in Milli-Q water.

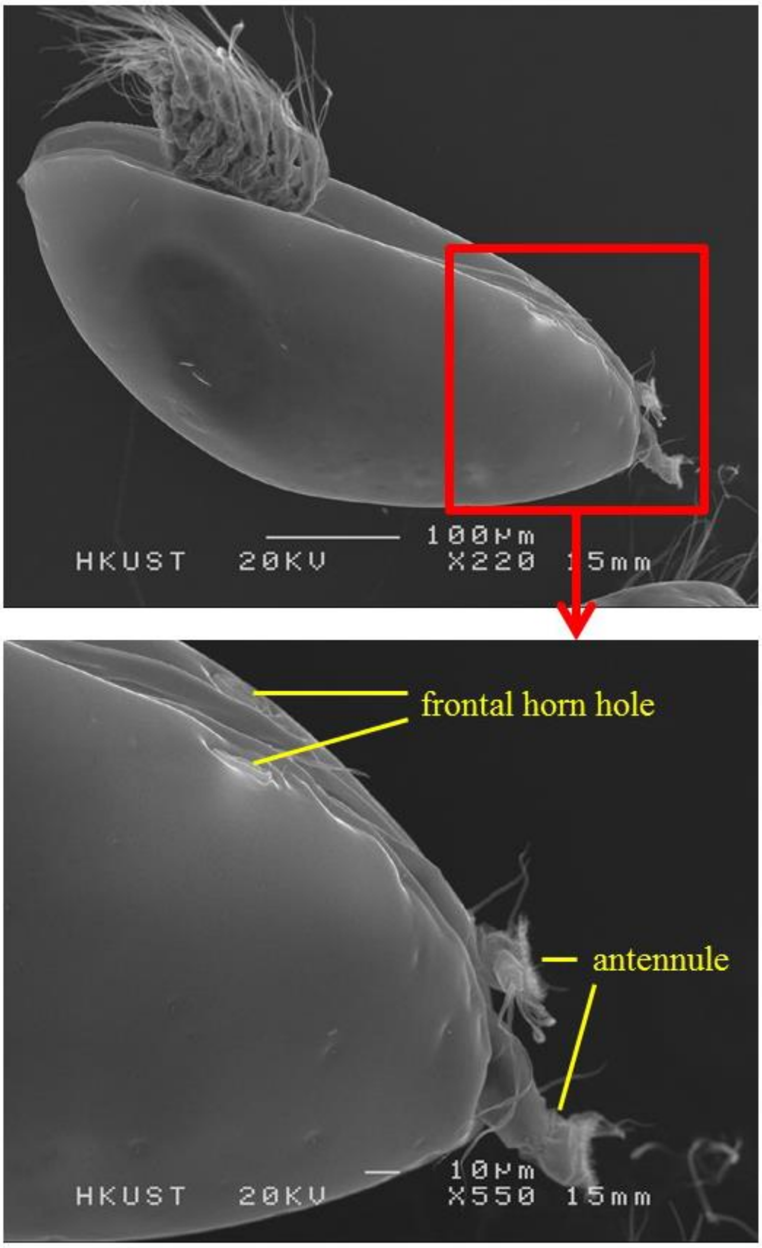

Supplementary Figure S8. A lateral view of a cyprid. In this position, the paired frontal horn holes could contact the underlying surface while the cyprids were exploring or climbing on a surface.

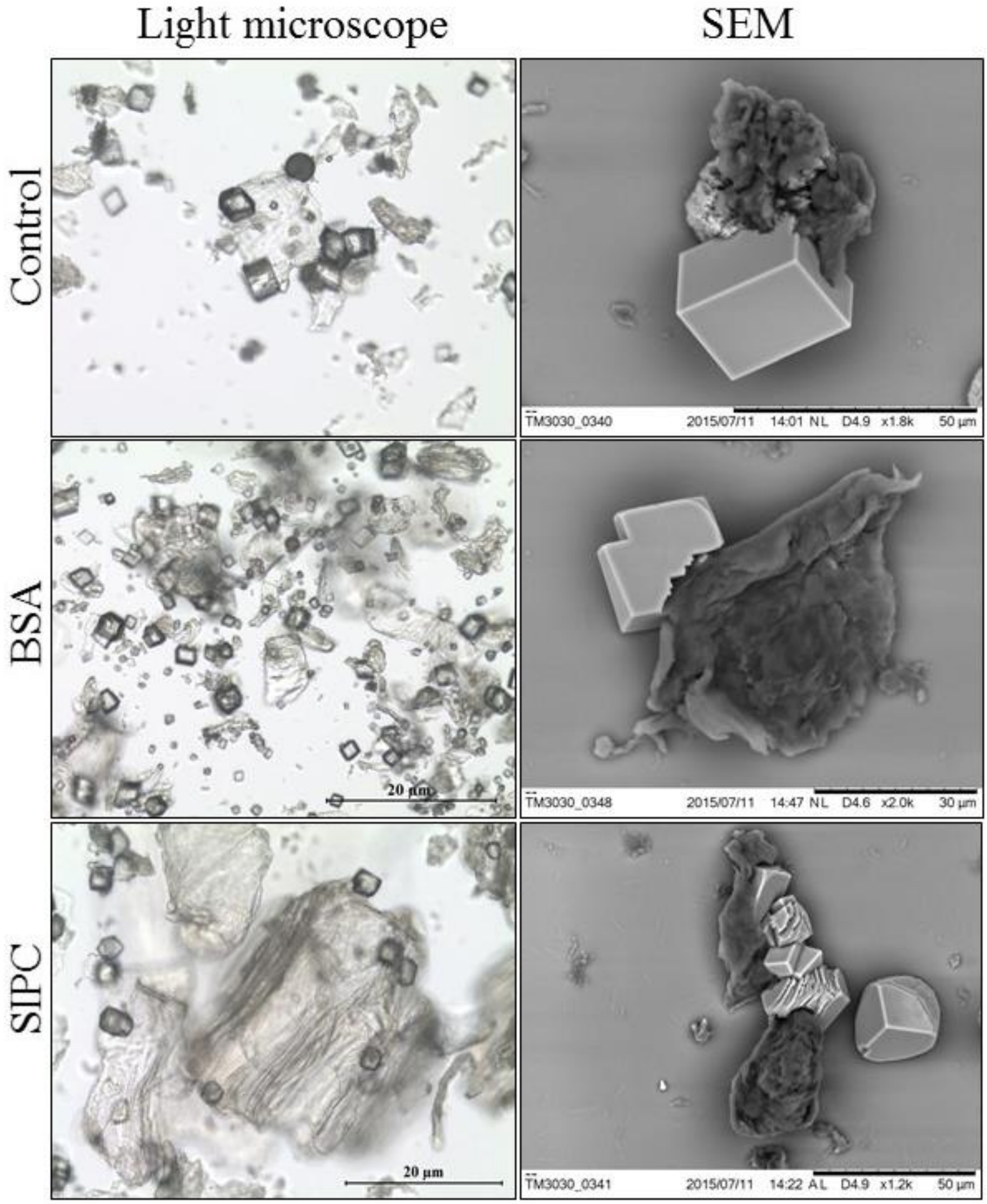

Supplementary Figure S9. $CaCO_3$ crystals were formed on chitin fragments in the presence/absence of BSA/recSIPC.

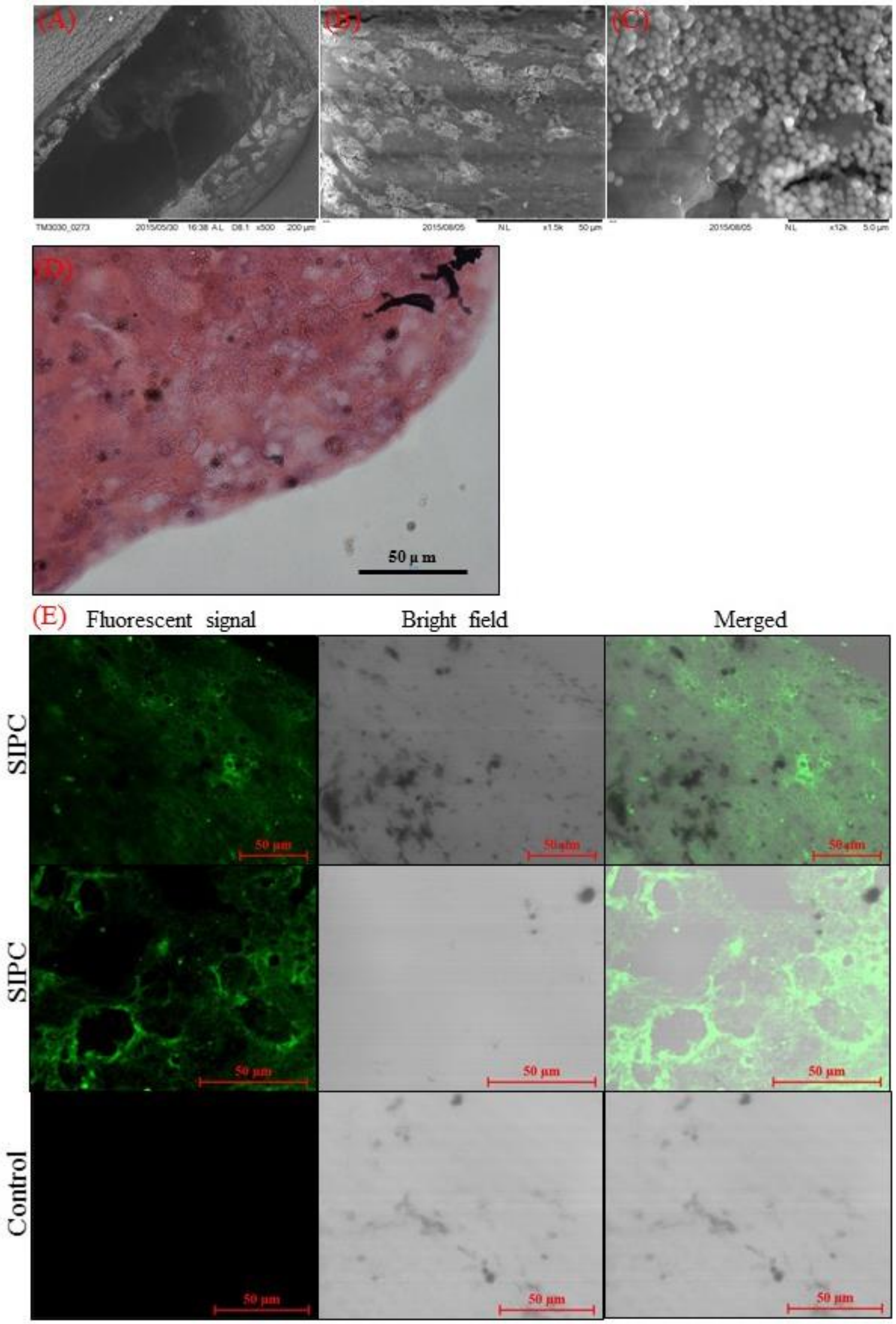

Supplementary Figure S10. SIPC, chitin and newly formed crystals were observed in the

membrane attached to the surface of channels inside barnacle shells. (A) SEM image showing the membrane inside a barnacle shell. (B). A membrane was separated from a barnacle shell and then placed on a glass slide. (C). Highly magnified SEM pictures showing newly formed crystals. (D). Hematoxylin and eosin (HE) staining revealing a network structure that is believed to be chitin in the membrane. (E). Positive immunostaining for SIPC at the membrane.

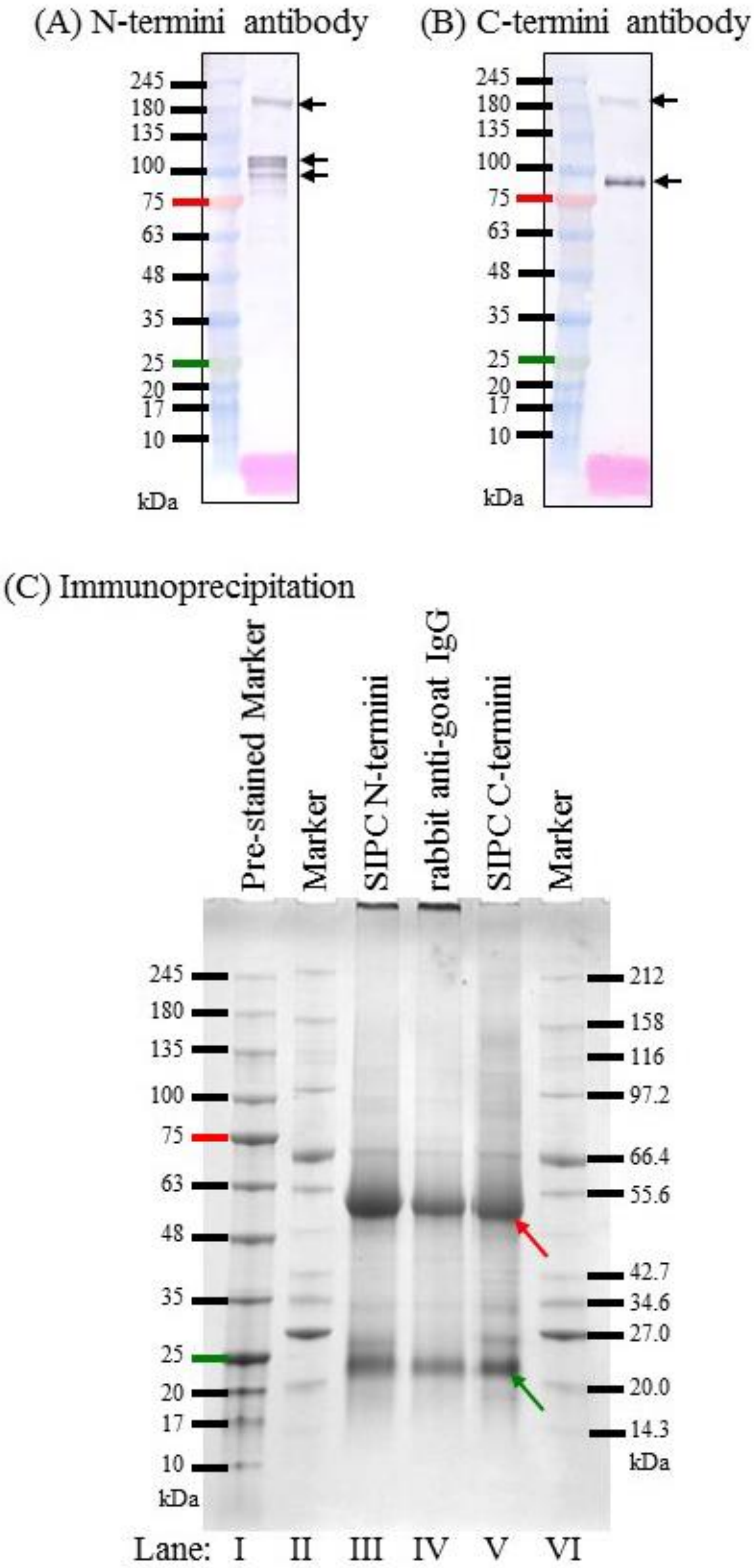

Supplementary Figure S11. Effectiveness of and specificity tests for the produced SIPC

antibodies. As previously reported, SIPC degraded into 3 subunits in barnacles, corresponding to 76, 88, and 98 kDa, due to unknown reasons. In the present study, 10 μg of total protein extracted from adult barnacles using 8 M urea were separated in a 4-20% gradient PAGE gel and then transferred onto a PVDF membrane. Western blot was performed using the SIPC antibodies. The secondary antibody was linked to HRP and the signals were revealed using a DAB (3,3-diaminobenzidine) substrate kit (Boster, Wuhan, China). (A). The antibody against SIPC N-termini detected 3 bands on the PVDF membrane (approximately 88, 98 and 200 kDa). (B). The antibody against the SIPC C-terminus detected 2 bands on the PVDF membrane (approximately 76 and 200 kDa). (C). Immunoprecipitation was conducted by incubating 40 μl (~10 μg) of antibody, 2 mg of total protein extracted from cyprids and 10 μl of A/G agarose beads (Sangon, Shanghai, China) at 4°C for 4 hours. The protein that was bound to the beads was separated in a 4-20% SDS PAGE gel and stained using Coomassie blue G250. No clear bands corresponding to SIPC were observed. However, after the gel pieces (from 75 to 200 kDa) was excised and digested with trypsin, the subsequent mass spectrometry analysis revealed the presence of SIPC in the treatments with SIPC antibodies but not in the control (rabbit anti-goat IgG antibody). The red and green arrows indicate the heavy and light chain of the antibody, respectively. Overall, Western blot and immunoprecipitation analysis results suggest that our antibodies are effective and specific to SIPC.

Supplementary Movie S1. A 3D view of SIPC-immunoreactive signals in a deposit left by cyprids.

Supplementary Movie S2. A 3D view of SIPC-immunoreactive signals in decalcified shell matrix sections.

Supplementary Movie S3. Crystals formed with recSIPC were more stable than crystals formed with BSA or the control. Crystals on coverslips were soaked in Milli-Q water for 60 hours. The crystals were then observed under an inverted microscope (Olympus XI51). The video was obtained using a Cannon 700D digital camera through an eyepiece. When a water current was generated with a pipette, the crystals formed in the control or BSA treatment detached from the surface and flowed with the current (Section I and II). However, the crystals formed with recSIPC remained attached to the surface (Section III).

Supplementary Movie S4. A cyprid "walking" on a surface. Cyprids were placed to a 24-well tissue culture plate and observed using an inverted microscopy (Olympus XI51). The ventral area of the frontal horn holes (indicated in Fig. S8) could touch the surface while the cyprids were "walking".
